# A text mining approach to categorize patient safety event reports by medication error type

**DOI:** 10.1038/s41598-023-45152-w

**Published:** 2023-10-26

**Authors:** Christian Boxley, Mari Fujimoto, Raj M. Ratwani, Allan Fong

**Affiliations:** 1 MedStar Health National Center for Human Factors in Healthcare, 3007 Tilden St., NW Suite 6N, Washington, DC 20008 USA; 2 MedStar St. Mary’s Hospital, Leonardtown, USA; 3 Georgetown University School of Medicine, Washington, USA

**Keywords:** Health services, Computational models, Data mining, Machine learning

## Abstract

Patient safety reporting systems give healthcare provider staff the ability to report medication related safety events and errors; however, many of these reports go unanalyzed and safety hazards go undetected. The objective of this study is to examine whether natural language processing can be used to better categorize medication related patient safety event reports. 3,861 medication related patient safety event reports that were previously annotated using a consolidated medication error taxonomy were used to develop three models using the following algorithms: (1) logistic regression, (2) elastic net, and (3) XGBoost. After development, models were tested, and model performance was analyzed. We found the XGBoost model performed best across all medication error categories. ‘Wrong Drug’, ‘Wrong Dosage Form or Technique or Route’, and ‘Improper Dose/Dose Omission’ categories performed best across the three models. In addition, we identified five words most closely associated with each medication error category and which medication error categories were most likely to co-occur. Machine learning techniques offer a semi-automated method for identifying specific medication error types from the free text of patient safety event reports. These algorithms have the potential to improve the categorization of medication related patient safety event reports which may lead to better identification of important medication safety patterns and trends.

## Introduction

Patient safety reporting systems provide a mechanism for healthcare provider staff, including frontline clinicians, nurses, and technicians to report patient safety errors and concerns^1^. While there are several categorization schemes for defining the types of errors that are reported, patient safety errors range from events where no harm occurs to the patient (e.g., “near misses” or “close calls”) to events in which patients are harmed (e.g., adverse events)^2^. Reporting systems vary by site; however, reports are typically composed of “structured data” and a free text description of the actual safety issue.

The promise of reporting systems is that they have the potential to dramatically improve the safety and quality of care by exposing possible vulnerabilities in the care process by documenting information on near miss and adverse events. Many provider organizations have promoted use of these systems, and while there are still barriers to reporting, many clinicians enter reports and provider organizations are amassing large databases^1,3–7^. Effectively analyzing these events has the potential for new insight as to where safety hazards reside^8^. There is also the opportunity to combine data across different provider organizations to identify patterns that may not be visible by looking at data from a single organization. Patient safety organizations (PSOs), which are legally secure environments for analyzing safety data, provide an opportunity to identify these broader trends.

While most organizations manually review their most significant cases that involve patient harm and conduct a root cause analysis or other review technique, this makes up a very small percent of the total number of reports^9^. For many organizations the number of reports has grown to tens of thousands and even hundreds of thousands, and for PSOs, the number of reports can be in the millions. The majority of safety reports are unanalyzed and recognized safety hazards that have not yet reached the level of patient harm go undetected by patient safety and risk analysts because they are buried in the large number of patient safety event (PSE) reports. This is a major shortcoming given that clinicians are taking the time to report, and the data are available.

There is an opportunity to apply machine learning techniques to improve the analysis of PSE reports so that these data can be used more effectively to identify patient safety patterns and trends so that interventions can be developed to address these trends. The objective of this study is to examine whether natural language processing (NLP) can be used to better categorize PSE reports, with a focus on medication related reports and categorization of these reports by medication error type.

## Background

### Medication safety event reports

When looking at the composition of PSE report databases, medication safety events are often the most frequently reported patient safety report type and are often associated with the greatest harm to patients^10^. Consequently, improving the analysis of these reports through a semi-automated approach will likely have significant impact given the volume of reports that may need to be reviewed and acted upon. A first step in analyzing medication related PSE reports is to determine the type of medication error described in the report. Semi-automatically categorizing medication related reports into the appropriate medication error type is a natural place to apply computational techniques given the importance of understanding the type of medication error and that well-defined medication error categories already exist.

### Natural language processing (NLP) as an automatic method to categorize reports

At a high level, NLP is a probability based method to detect patterns in text and to categorize based on these patterns^11^. NLP has been used extensively in healthcare to analyze clinical documents to identify specific healthcare conditions, identifying drugs, mining the electronic health records, as well as several other applications^12–16^. To realize the tremendous value of NLP to the analysis of PSE reports it is important to understand the nature of a PSE report. Each report generally contains structured information such as the time and site of occurrence (e.g., emergency department, blood bank), role of the participants (e.g., physician, nurse, technician), patient demographics (e.g., age, gender), as well as a classification of the severity and type of event (e.g., death, harm, near-miss). The type of event is a general category label such as “fall”, “medication”, “lab”, etc. These event categories can vary by institution and can even vary within institution depending on whether the same reporting system is being used. In addition to the structured data elements, the reports also include an unstructured free-text field in which the reporter can provide a narrative describing the safety event in greater detail. Here, reporters can contextualize safety events and near misses by documenting contributing factors, the circumstances surrounding the event, and other information not captured in the structured fields. These free-text fields provide incredible value to organizations looking to minimize system-based risks. Given that the PSE reports generally have rich free text responses, various NLP techniques to extract health and medical concepts, relationships, negations, tense, and causation lend themselves for expediting the analysis of large numbers of reports by removing the need for analysts to read all reports^17,18^. Previous work has demonstrated how NLP techniques can be implemented into workflows to improve patient safety^19^. With the growing focus on medication safety^20^, it is important to understand how similar techniques can be used to understand and prevent medication errors.

### Medication error type categories

A commonly used taxonomy to describe medication errors is the National Coordinating Council for Medication Error Reporting and Prevention (NCC MERP) taxonomy^21^. The taxonomy is composed of several different categories and the specific type of medication error (e.g., wrong drug, wrong rate, etc.) is one of the most commonly used parts of the taxonomy. Nearly all medication related PSE reports are categorized into these medication error types somewhere in the reporting and analysis process. This categorization may be performed by the reporter when entering the port and/or by the analyst when reviewing reports and attempting to make sense of the reports. We sought to semi-automatically categorize PSE reports into the appropriate medication error type to improve the analysis process.

## Methods

### Data source

**Figure 1:**
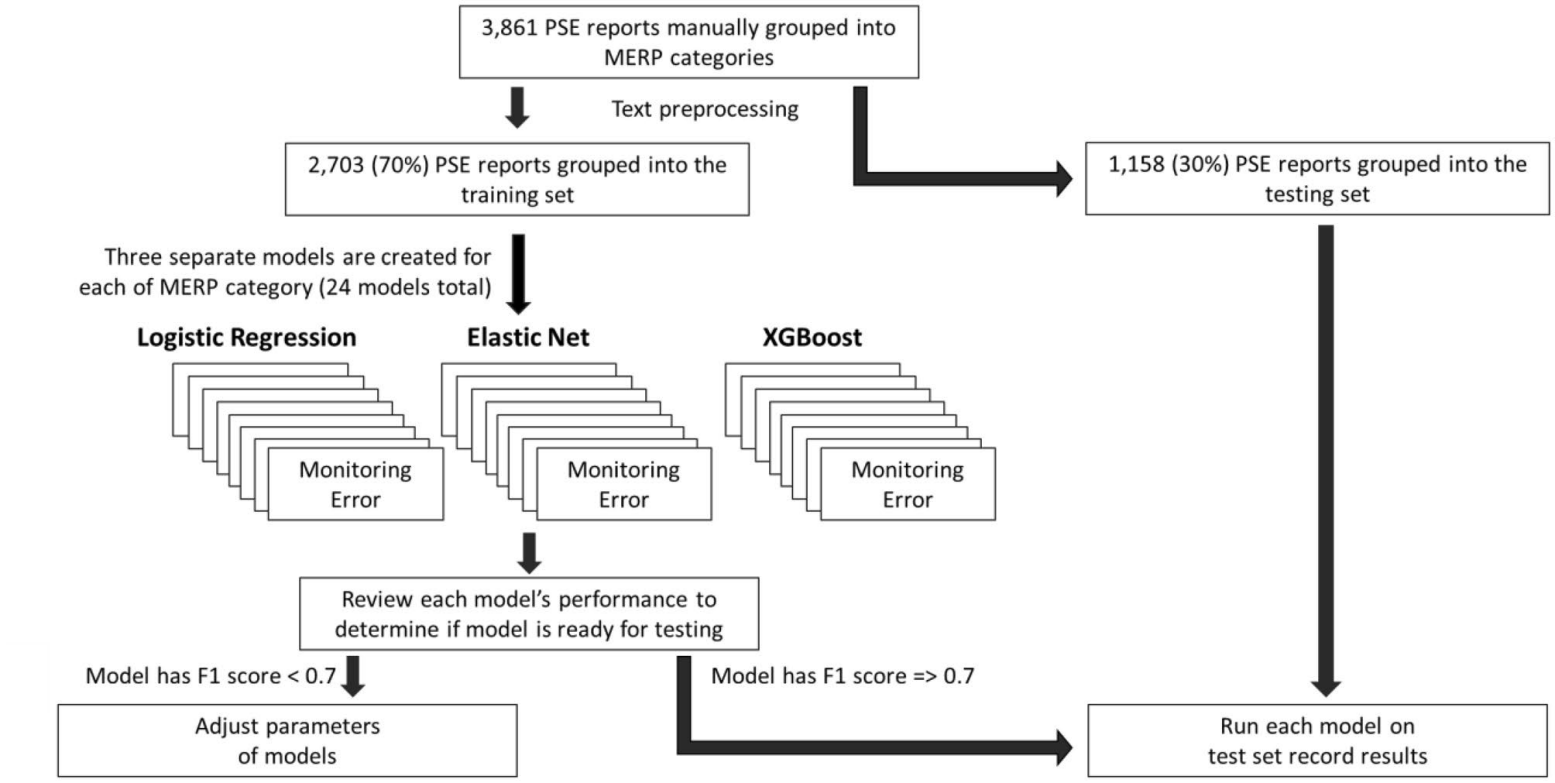
Flow diagram for how models were developed and tested to classify PSE reports in MERP categories.

This paper uses 3,861 PSE reports from a ten-hospital healthcare system in the mid-Atlantic region of the United States. Hospitals from this system range from large, academic hospitals found in urban centers to smaller community hospitals in rural settings. The patient population is diverse in terms of race/ethnicity, gender, age, and health condition resulting in a generalizable data set. Structured fields in the reporting system include department, general event type, specific event type, and severity level. These reports were previously annotated by subject matter experts (a pharmacist and patient safety analysts) using a consolidated MERP framework^22^. We use these annotations for our model training and testing, Fig. 1.

### MERP categories

For this study, the original 14 subcategories of “Error Type” found in the standard taxonomy of medication errors from the NCC MERP were modified into eight categories. During manual review of the reports, we determined limitations in the free text of some reports made it impossible to distinguish some of the similar and related categories without making too many assumptions (e.g., wrong strength and wrong concentration). This led to our use of a consolidated MERP framework and highlights the challenges with overlapping concepts in MERP categories, especially for complex free text narratives. Each report could fall into zero, one, or multiple MERP categories including: (1) wrong drug, (2) wrong time, (3) wrong strength or concentration, (4) wrong dosage form or technique or route, (5) improper dose/dose omission, (6) wrong rate, (7) wrong patient, and (8) monitoring error, defined in Table 1. Most reports were categorized into one or two MERP categories but could be categorized into as many as six, Table 2.

**Table 1:** Breakdown of MERP categories for 3,861 PSE reports. Each report could be grouped into zero, one, or multiple categories.

MERP categories	Definition	Number of reports
Coding taxonomy and definitions^21^		
Wrong drug	Ordered, dispensed, or administered a medication different from what was intended or gave a drug when no drug was intended	1982
Wrong time	Administration outside a predefined time interval from its scheduled administration time, as defined by each health care facility	1677
Wrong strength or concentration	Incorrect medication strength/concentration was ordered, dispensed, or administered	1453
Wrong dosage form or technique or route	Combined from wrong dosage form, wrong technique, and wrong route of administration	1107
Improper dose/dose omission	Failure to order, dispense, or administer a dose as intended. Includes an overdose, underdose, extradose, or duplicate therapy due in some part to an incorrect duration, strength/concentration, dosage form, rate, time, or frequency	1102
Wrong rate	Medication administered too fast or slow	323
Wrong patient	Medication was ordered, dispensed, or administered to patient different from what was intended	152
Monitoring error	Drug-drug interactions or drug allergy issues that were not identified	144

**Table 2:** The number of MERP categories for each of the 3,861 reports after manual annotation.

Number of MERP categories	Number of reports
0	84
1	1411
2	1181
3	707
4	361
5	100
6	17

### Experimental pipeline

#### Text preprocessing and feature selection

One researcher automated the preprocessing of the free text from the 3,861 PSE reports. The goal of this preprocessing step is to reduce noise and make the text ready to feed into our natural language processing models. Numbers and punctuations were removed, and all the free text was lower-cased using an automated computer program. We used unigrams (i.e., single words like “patient”, “medication”, or “prescription”), bigrams (i.e., consecutive word pairs like “patient allergy”, “medication dose”, or “prescription written”), and trigrams (i.e., consecutive word triplets like “patient allergy overlooked”, “medication dose incorrect”, or “prescription written late”) terms to ensure we were capturing strings of words that were particularly meaningful. We excluded terms that appeared in greater than 99 percent and less than one percent of reports. Words that appear in 99 percent of reports are often context specific stop words, such as ‘the’, ‘a’, and ‘patient’ and do not help model performance. Words that appear in less than one percent (often times proper names) typically will introduce more noise into the modeling process. This filtering process is a common step in text preprocessing to improve model performance^23^. Term frequency-inverse document frequency (TFIDF) was used to create a ngram (unigram, bigram, and trigram) feature vector for each report.

#### Model development and testing

For each MERP category, we evaluated three algorithms to predict whether a report did or did not fall into the category based on the report’s free text. The algorithms used for each MERP category were: (1) Logistic regression, (2) Elastic net, and (3) XGBoost. Logistic regression was chosen as an interpretable model for binary dependent variables. Elastic net was chosen as it expands upon logistic regression by combining the L1 and L2 penalties of lasso and ridge regression methods and minimizing the loss^24^. XGBoost was selected because it implements the gradient boosting decision tree algorithm which sequentially adds new models together to make predictions while minimizing loss^25^.

A One-vs-rest (OvR) approach was taken for each MERP category. OvR is a common heuristic method used when multiple classes (e.g., multiple MERP categories) are present in a classification problem. Using OvR, we can change our multi-class classification problem into multiple binary classification problems allowing the use of algorithms like logistic regression and more interpretable models.

The models were trained and validated on 70 percent of the original 3,861 reports using fivefold cross validation. While there is no hard rule for the percentage and number of CV folds, 80 percent for training and validation and using fivefold CV is a common practice when building machine learning models^26–28^. We choose a slightly lower percentage for training and validation because of the data imbalance and to avoid overfitting while reserving enough data to test. The hyperparameters of the elastic net model were tuned with cross-validation. Bayesian optimization was utilized to optimize the hyperparameters of the XGBoost. Each model was then tested on the held-out testing dataset (i.e., the remaining 30 percent of reports). Our metrics included a confusion matrix, precision, recall, specificity, F1 score, area under the curve—receiver operating characteristic curve (AUC-ROC), precision-recall and receiver operating characteristic curve (PR-ROC), and accuracy.

A confusion matrix describes the complete performance of the model by outputting (from left to right, top to bottom): true positives (TP), false positives (FP), true negatives (TN), and false negatives (FN). Precision measures the number of correct positive predictions by dividing the number of positive results predicted by the classifier calculated as: (TP)/(TP + FP). Recall (ie sensitivity) measures the number of actual positives that were correctly identified by calculating: (TP)/(FN + TP). Specificity measures the number of actual negatives that were correctly identified by calculating: (TN)/(TN + FP). F1 score is the harmonic mean between precision and recall calculated as: 2 * (1/((1/precision) + (1/recall)). AUC-ROC is a measurement that represents how well the model distinguishes between classes. PR-ROC is a measurement that represents the trade-off between the true positive rate (precision) and the positive predictive value (recall). Lastly, accuracy is the percentage of correct predictions.

Lastly, we used the *gain* metric to identify the five most important features in the best performing model for each MERP category. *Gain* implies the relative contribution of the corresponding feature to the model calculated by taking each feature’s contribution for each tree in the model. A higher value of this metric when compared to another feature implies it is more important for generating a prediction.

The study presented no risk to animal and/or animal subjects and was reviewed by the Institutional Review Board at MedStar Health Research Institute. All experimental protocols were approved by the Institutional Review Board at MedStar Health Research Institute. This research was performed in accordance with relevant guidelines and regulations.

## Results

### Model performance

Model performances for the three different algorithms are shown in Table 3. Performance scores were generally lowest when using logistic regression and highest when using XGBoost. When using logistic regression, the average F1 score across MERP categories was 0.54 (standard deviation of 0.26). For elastic net, the average F1 score across MERP categories was 0.59 (standard deviation of 0.23). The average F1 score across MERP categories was 0.72 (standard deviation of 0.15) when using XGBoost. Performance across MERP categories is also shown in Table 3. The ‘Wrong Drug’ category performed best across all three algorithms while the ‘Wrong Patient’ category generally had the lowest performance metrics.

**Table 3:** Performance of the logistic regression, elastic net, and XGBoost algorithms across the eight MERP categories.

			Logistic regression							ElasticNetCV							XGBoost						
MERP Category	# of true positives (training)	# of true positives (testing)	Precision	Recall	Specificity	F1	AUCROC	PR-ROC	Accuracy	Precision	Recall	Specificity	F1	AUCROC	PR-ROC	Accuracy	Precision	Recall	Specificity	F1	AUCROC	PR-ROC	Accuracy
Wrong Drug	1371	611	0.88	0.81	0.87	0.84	0.92	0.91	0.84	0.86	0.89	0.88	0.87	0.92	0.92	0.86	0.92	0.91	0.91	0.92	0.95	0.94	0.91
Wrong Time	1170	507	0.70	0.67	0.77	0.68	0.82	0.77	0.73	0.71	0.57	0.74	0.63	0.80	0.74	0.71	0.72	0.74	0.78	0.73	0.83	0.75	0.76
Wrong Strength or Concentration	1007	446	0.73	0.56	0.87	0.63	0.82	0.74	0.75	0.71	0.59	0.84	0.64	0.86	0.73	0.75	0.71	0.66	0.83	0.68	0.83	0.75	0.76
Wrong Dosage Form or Technique or Route	759	348	0.95	0.58	0.99	0.72	0.90	0.86	0.87	0.90	0.66	0.98	0.76	0.90	0.86	0.88	0.95	0.79	0.98	0.86	0.93	0.90	0.92
Improper Dose/Dose Omission	765	337	0.90	0.50	0.98	0.64	0.91	0.82	0.84	0.85	0.62	0.98	0.71	0.91	0.85	0.86	0.92	0.95	0.95	0.84	0.95	0.90	0.91
Wrong Rate	219	104	0.91	0.31	1.00	0.46	0.96	0.74	0.94	0.86	0.42	1.00	0.57	0.96	0.76	0.94	0.87	0.63	0.99	0.73	0.95	0.82	0.96
Wrong Patient	104	48	1.00	0.04	1.00	0.08	0.94	0.56	0.96	0.80	0.08	1.00	0.15	0.94	0.55	0.96	0.89	0.35	1.00	0.51	0.91	0.54	0.97
Monitoring Error	99	45	0.86	0.13	1.00	0.23	0.84	0.42	0.97	0.91	0.22	1.00	0.36	0.84	0.40	0.97	0.89	0.36	1.00	0.51	0.84	0.49	0.97

### Co-occurrence of MERP categories

754 out of 1,159 (65.1%) reports in our testing dataset were manually categorized into two or more MERP categories. In over 75 percent of reports categorized as ‘Wrong Drug’, the reports were also categorized as ‘Improper Dose/Dose Omission’ or ‘Wrong Time’. In addition, reports categorized as ‘Wrong Time’ co-occurred with ‘Improper Dose/Dose Omission’ or ‘Wrong Drug’ over two-thirds of the time, Table 4.

**Table 4:** MERP category prediction correlations using XGBoost.

	Predicted wrong drug	Predicted wrong time	Predicted wrong strength	Predicted wrong dosage form	Predicted improper dose	Predicted wrong rate	Predicted wrong patient	Predicted monitoring error
True wrong drug	558	371	191	85	112	43	8	11
True wrong time	353	374	145	85	191	45	5	9
True wrong strength	200	157	293	159	94	36	1	7
True wrong dosage form	162	113	176	276	79	12	0	8
True improper dose	237	207	79	63	271	19	9	7
True wrong rate	54	54	34	9	20	66	0	1
True wrong patient	18	26	2	3	15	0	17	0
True monitoring error	24	17	10	8	13	1	0	16

### Most important features within MERP categories

We used the *gain* metric to evaluate the five most important features (i.e., words) from each XGBoost model, Table 5. XGBoost models had on average the highest F1 score for each MERP category. The features for ‘Wrong Drug’, ‘Wrong Time’, and ‘Improper Dose/Dose Omission’ were generally verb or action words such as *entered* and *ordered*. ‘Wrong Strength or Concentration’ and ‘Wrong Rate’ were often measurement or units. ‘Wrong Dosage Form or Technique or Route’ forms like *tablet* and *extended release*. ‘Wrong Patient’ was about workflow actions and nouns around patient while ‘Monitoring Errors’ were around allergies and other monitoring of symptoms.

**Table 5:** The five most ‘important’ features when making predictions for each MERP category using XGBoost. Free text examples are lightly edited for clarity.

MERP categories	Highest importance features	Free text
Wrong drug	Entered; ordered; discontinued; order; orders	“During quality assurance, nurse found that the patient wass prescribed both levaquin and azithromycin concurrently (both taken together have a major interaction). Doctor called and **discontinued** the azithromycin.”
Wrong time	Removed; gave; prescription; order; ordered	“Patient had a lidocaine patch placed It was ordered to be **removed** 12 h later at pm and was never removed. Dayshift nurse the next day found dated lidocaine patch and removed. Medication was retimed accordingly.”
Wrong strength or concentration	mcg; ml; directions; gm; mg	“Ticagrelor (Brillinta) should be taken with no more than 81 mg aspirin. Patient received ticagrelor dose, then aspirin 325 mg ordered.”
Wrong dosage form or technique or route	Tablet; tablets, er, stable, tab	“Prescription was filled for a drug in capsule form instead of **tablets** that were prescribed. Claim was reserved, drug was returned to stock, and the correct drug form was filled.”
Improper dose/dose omission	Discontinued; missed; discontinue; given; briefly	“Physician incorrectly placed a bolus order- 500 mL of 0.45% NS…recommended that bolus dose should be dosed at 10–20 mL/kg. Physician **discontinued** the previous order and placed another order with appropriate weight-based dosing.”
Wrong rate	Rate; mlhr; fluid; renal; remained	“Medical Administration Record stated to give intravenous immune globulin at starting rate of 22 mL/min. Called pharmacy to verify rate and was told **rate** was incorrect. Medication reordered with correct **rate** of 22 mL**/hr**.”
Wrong patient	Wrong patient; brought; outpatient; realized; working	“Prescription for bedside entered under **wrong patient**; prescription was for apixaban 5 mg. Advised technician to correct.”
Monitoring error	Allergy; lovenox; symptoms; attending; plan	“Patient was ordered robotussin with codeine prn for cough. Patient has codeine listed as **allergy**.”

## Discussion

### Algorithm application in medication safety workflows

This study categorizes patient safety event reports into medication error categories and compares model performance in this large dataset across three different algorithms (e.g., logistic regression, elastic net, and XGBoost). Like previous work^29^, we find that our method saves time by programmatically processing reports and making themes in medication errors easier to uncover compared to manually reading through each report to group into MERP categories. In addition, the structured categories available to reporters are limited, and reporters do not always select the most appropriate categories. Our method bypasses the unreliability of the structured categories and groups reports by their free text.

Building off previous work, our analysis of the co-occurrence of MERP categories highlights the higher level of complexity when assigning multiple MERP categories to a report. Future work should develop a belief network to fully understand the correlation between MERP categories. Using the *gain* metric to determine feature importance allows a better understanding of the unique aspects of each type of medication errors.

There are several opportunities to apply natural language processing and machine learning techniques to improve medication safety. First, these algorithms could be integrated into reporting systems to guide the person entering the report to select a structured category that best aligns with the appropriate MERP category. This would serve to reduce inappropriate classifications and the labor-intensive recoding of reports. Second, the algorithms could be applied across all PSE reports, even non-medication reports, to identify patterns and trends in PSE report data. This is especially important for patient safety organizations and other stakeholders that are analyzing large datasets of safety event reports. Finally, patient safety committees that are looking for different patterns and trends in PSE report data may want to apply these algorithms to identify whether specific actions should be taken based on the emerging patterns.

MERP categories could be integrated with other structured categories in the reporting system such as medication names mentioned, departments, event date, etc. With the MERP categories identified, patient safety analysts could quickly identify specific medication errors related to a medication, hospital, or site. MERP categories could also be tracked and monitored over time. Future work should formally implement similar models into quality and safety workflow or develop more complex models to determine the benefit of these and similar models.

### Challenges and limitations

The voluntary nature of PSE reports often led to under reporting and should be used primarily to identify general themes but often cannot conclude causality. In addition, working with free text is difficult–especially the free text found in PSE reports. Often these reports include abbreviations, medical jargon, and misspellings that present challenges for analysis. These challenges can be seen in the MERP categories that performed poorly across the three algorithms. The two MERP categories that performed the worst also had the smallest sample sizes suggesting that training these models with limited datasets can also lead to poor performance in certain categories. Though difficult with datasets such as PSE reports, future work should strive to work with more balanced datasets with equal representation across all MERP categories to ensure consistent model performance.

Further in complicated cases, incident reporters and human annotators could categorize error types based on their clinical experience, reflection of occupational responsibilities, and expectation. However, such human or personal perceptions were not always expressed in sentences that our current model can use to categorize the error types. This presents an opportunity for more sophisticated machine learning techniques to be used in future analyses of medication errors in PSE reports. Future work should consider leveraging large language models like GPT (Generative Pre-trained Transformer) and BERT (Bidirectional Encoder Representations from Transformers) and comparing performance to the three algorithms used in this study. Because of the previously noted abbreviations, medical jargon, and misspellings commonly found in these reports, a hybrid or human in the loop approach to developing machine learning-based models should also be considered to mitigate model shortcomings. Furthermore, integrating these models into an interactive visualization allows the clinical staff to gain insights as well as provide feedback and corrections to update the model results in near-real time.

## Conclusion

NLP techniques may offer a semi-automated method for identifying specific medication error types from the free text of PSE reports. The analysis and categorization of patient safety event reports often require expert review and can be a time-consuming process. In this case report, we applied various NLP techniques to recategorize medication patient safety events into specific workflow related categories. These categories provide insights into system and workflow processes that might require additional attention.

## Data Availability

The datasets generated and/or analyzed during the current study are not publicly available because they contain sensitive patient health information. However, deidentified data are available from the corresponding author on reasonable request.
